# The authors reply: Letter on: “ Sarcopenia and its association with falls and fracturesin older adults: A systematic review andmeta‐analysis” by Zhang et al.

**DOI:** 10.1002/jcsm.12521

**Published:** 2020-01-05

**Authors:** Suey S.Y. Yeung, Martijn W. Heymans, Andrea B. Maier

**Affiliations:** ^1^ Department of Human Movement Sciences, @AgeAmsterdam, Faculty of Behavioural and Movement Sciences, Amsterdam Movement Sciences Vrije Universiteit Amsterdam The Netherlands; ^2^ Department of Medicine and Aged Care, @AgeMelbourne, The Royal Melbourne Hospital The University of Melbourne City Campus, Level 6 North, 300 Grattan Street Parkville Victoria 3050 Australia; ^3^ Department of Epidemiology and Biostatistics, Amsterdam Public Health Research Institute VU University Medical Center Amsterdam The Netherlands

Thank you for the comments to our recent article “Sarcopenia and its association with falls and fractures in older adults: A systematic review and meta‐analysis”.^1^

**Figure 1:**
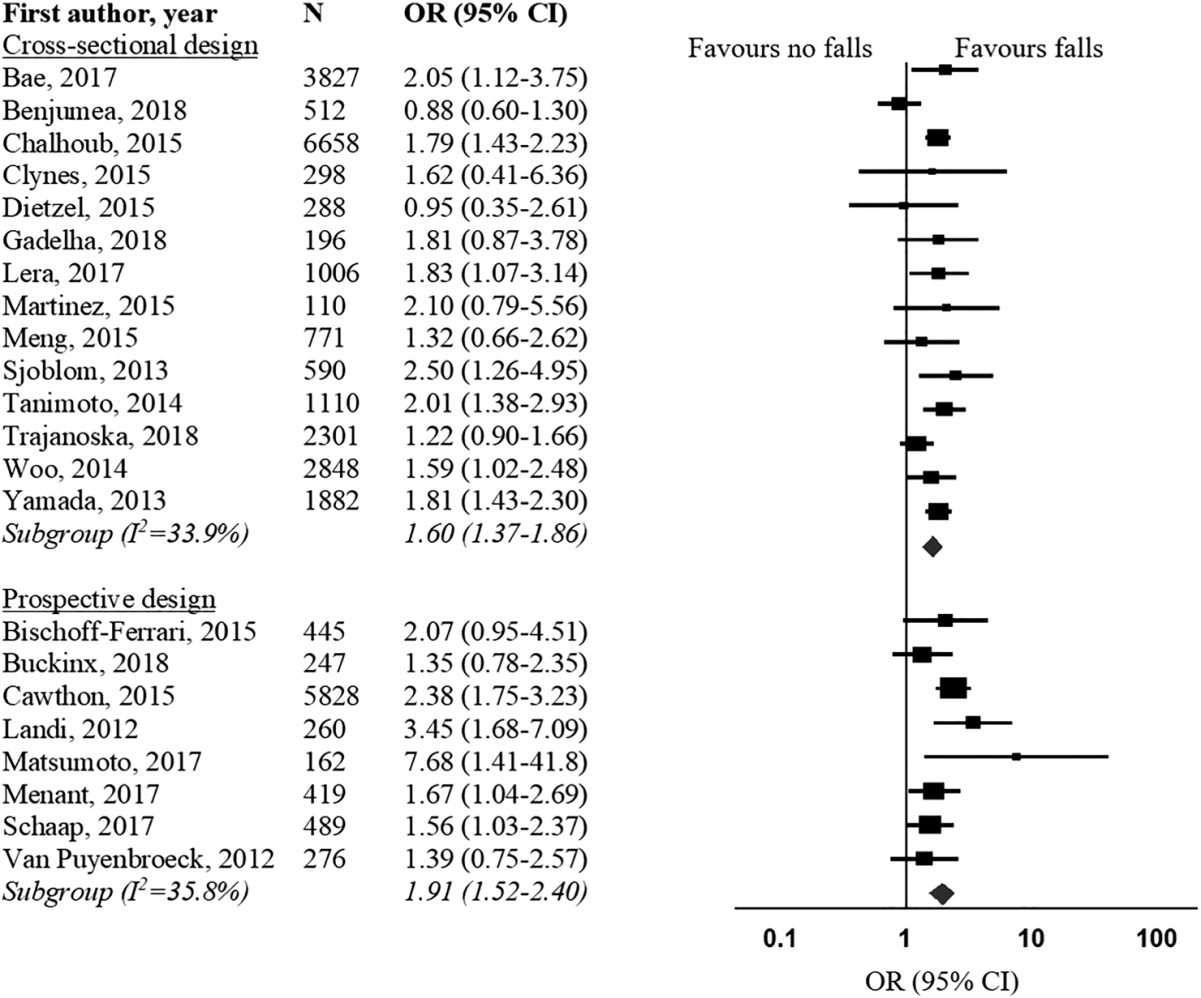
Forest plot of odds ratio for falls in sarcopenic individuals vs. non‐sarcopenic individuals

**Figure 2:**
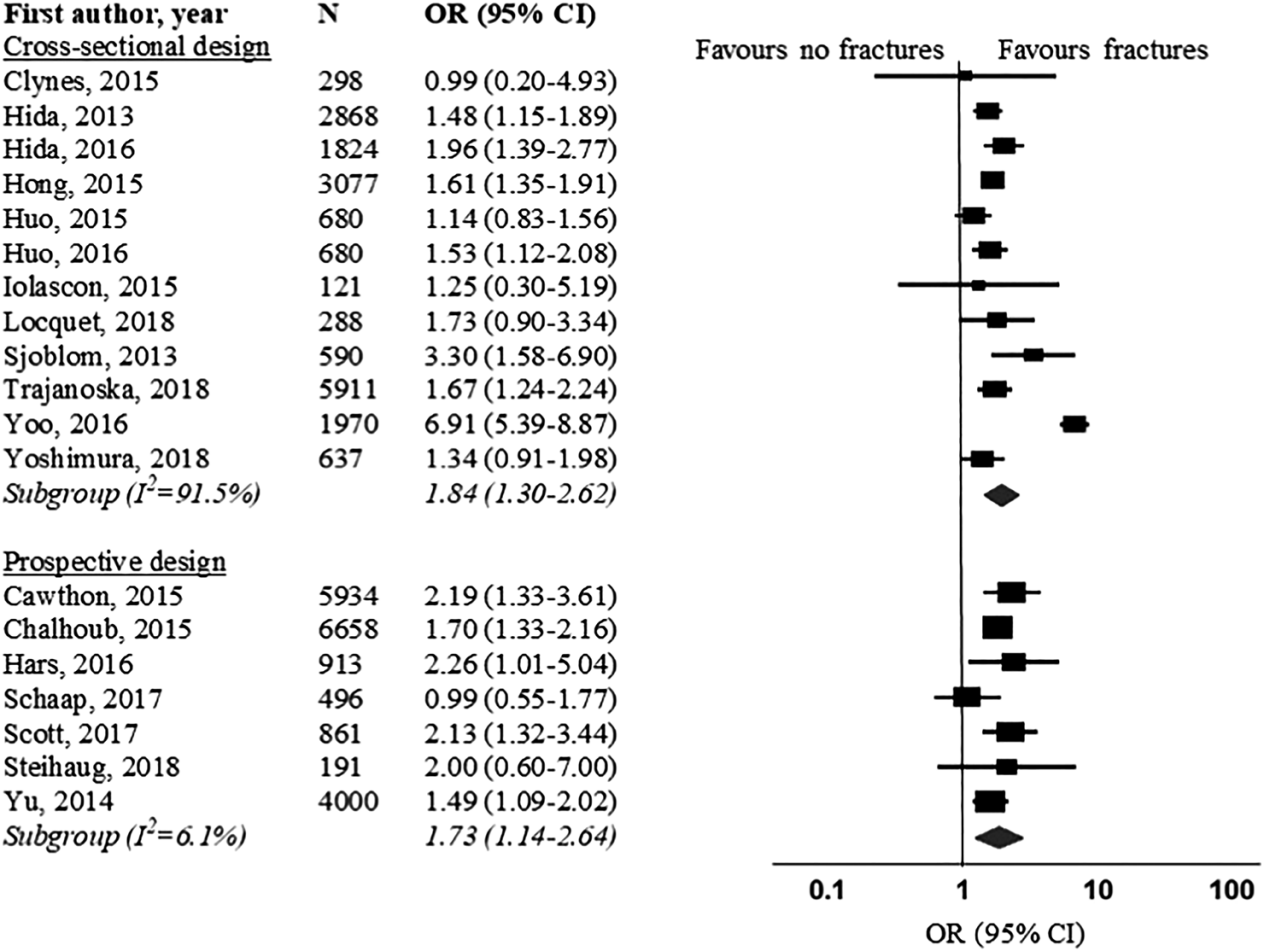
Forest plot of odds ratio for fractures in sarcopenic individuals vs. non‐sarcopenic individuals

Firstly, as mentioned in our article, studies were excluded from the meta‐analysis if an odds ratio (OR) could not be calculated because of insufficient data, or confidence intervals were not presented. Therefore, we were not able to include the studies of Cawthon,^2^ Schaap,^3^ and Henwood,^4^ as hazard ratios^2^, ^3^ or risk ratios^4^ were reported. These measures are not interchangeable with ORs^5^, ^6^ but can be converted if information about the baseline risk is available.^7^ We agree that the above‐mentioned studies are valuable; therefore, we contacted the authors to obtain the data needed to compute ORs. Two authors of the three studies responded, which allowed us to include those studies in the meta‐analysis.^2^, ^3^ Both were prospective studies examining the association between sarcopenia with falls and fractures. ORs reported in our original article^1^ did not change significantly after inclusion of these studies (falls prospective studies: pooled OR 1.91, 95% CI 1.52–2.40, *P* < 0.001, *I* ^2^ = 36%; fractures prospective studies: pooled OR 1.73, 95% CI 1.14–2.64, *P* = 0.011, *I* ^2^ = 6%) (Figures 1 and 2).

**Figure 3:**
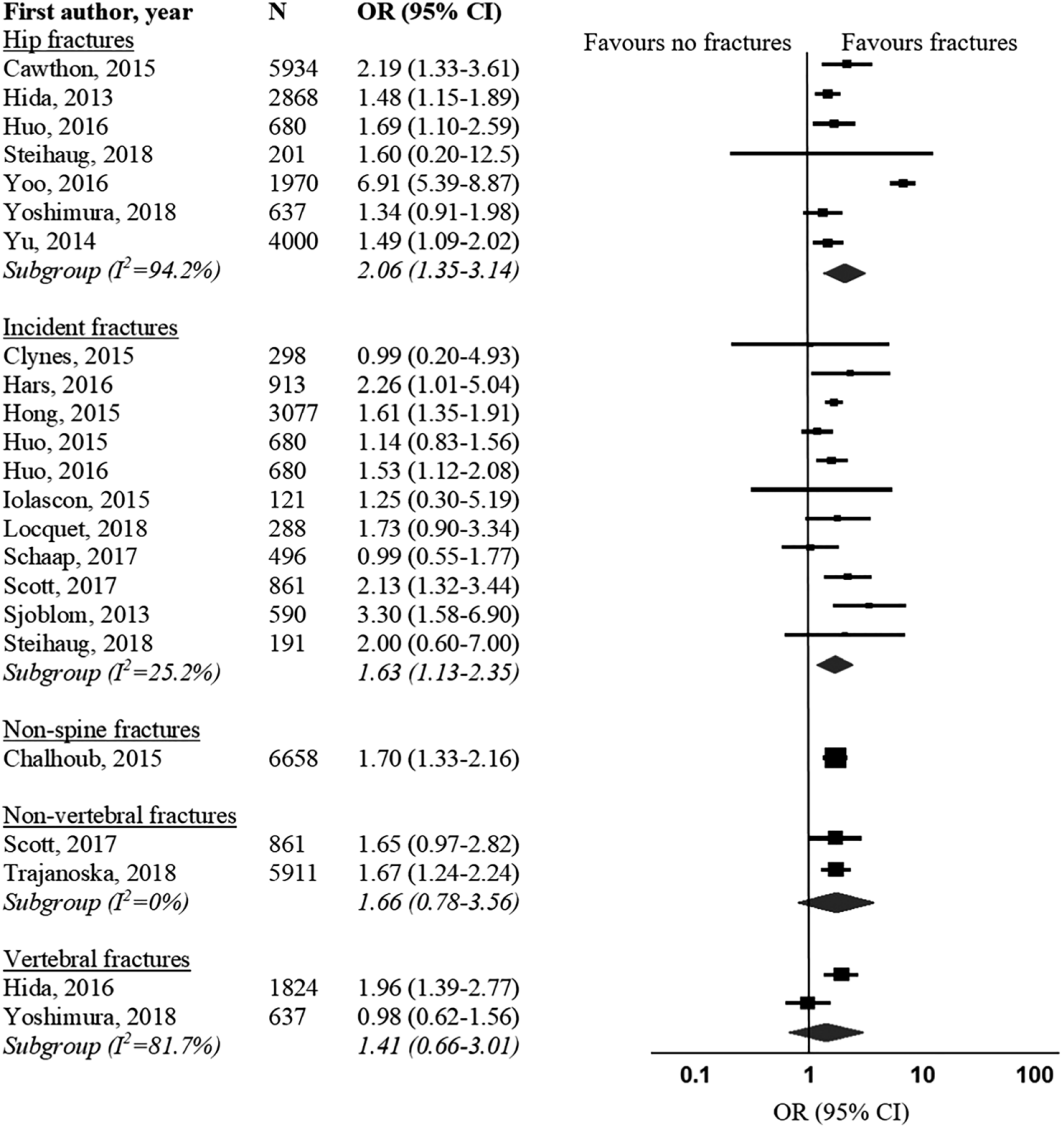
Forest plot of odds ratio for fractures in sarcopenic individuals vs. non‐sarcopenic individuals, stratified by fracture types

Secondly, Zhang *et al*. suggested to take the types of fracture into consideration when conducting the meta‐analysis of the association between sarcopenia and fractures. We agree with the authors that fracture sites vary significantly in their risk profiles^8^ and appreciate the subgroup analysis performed by them. However, we have updated the subgroup analysis because (i) some studies reported the association between sarcopenia with more than one type of fracture, and (ii) we obtained additional data from Cawthon^2^ and Schaap^3^ to compute ORs. Our subgroup analysis showed a significant association between sarcopenia and hip fractures (OR 2.06, 95% CI 1.35–3.14, *P* = 0.001, *I* ^2^ = 94%) and incident fractures (OR 1.63, 95% CI 1.13–2.35, *P* = 0.009, *I* ^2^ = 25%), but the association was insignificant for non‐vertebral fractures (two studies) (OR 1.66, 95% CI 0.78–3.56, *P* = 0.190, *I* ^2^ = 0%) and vertebral fractures (two studies) (OR 1.41, 95% CI 0.66–3.01, *P* = 0.373, *I* ^2^ = 82%) among older adults (Figure 3).

Finally, Zhang *et al*. raised an important point regarding the number of falls. As each fall is associated with a risk of injury, functional decline, and loss of autonomy, the risk profile of recurrent fallers is not equivalent to that of single fallers.^9^, ^10^ Of the 22 studies included in our meta‐analysis, 20 studies reported at least one fall (≥1) as outcome, and two studies reported recurrent falls (≥2) as outcome.^2^, ^3^ We acknowledge the limitation of our article that a subgroup analysis regarding the number of falls cannot be performed owing to insufficient data.
